# On the move: exploring Inuit and non-Inuit health service providers’ perspectives about youth, family and community participation in care in Nunavik

**DOI:** 10.1186/s12913-021-06058-3

**Published:** 2021-01-28

**Authors:** Sarah Louise Fraser, Louise Moulin, Dominique Gaulin, Jennifer Thompson

**Affiliations:** 1 School of Psychoeducation, University of Montreal, Pavillon Marie-Victorin, C. P. 6128, succursale Centre-ville, Montréal, Québec H3C 3J7 Canada; 2 Centre de Recherche en Santé Publique (CReSP), University of Montreal and CIUSSS du centre Sud-de-l’Île de Montreal, Montreal, Canada; 3 School of Social Work, University of Montreal, Montreal, Canada

**Keywords:** Inuit, Participation, Nunavik, Youth, Families

## Abstract

**Background:**

Literature about participation in health and social services suggests that youth, and more specifically Indigenous youth, are difficult to engage within health and social services. Youth are less likely to access services or to actively participate in decision-making regarding their personal care. Service providers play a crucial role in engaging youth based on the ways in which they seek, establish, and maintain relationships with youth and their families. The way in which providers engage with youth will depend on various factors including their own perceptions of the roles and relationships of the various people involved in youth’s lives. In this article, we analyze health and social service providers’ perspectives, experiences and expectations regarding the roles of Indigenous youth, families and community in care settings in Nunavik, Quebec.

**Methods:**

Using a snowball sampling approach, we recruited 58 interview participants (39 non-Inuit and 19 Inuit), including psychiatrists, general practitioners, nurses, social workers, school principals, teachers, student counsellors, representatives of local committees, and police officers. The interviews focused on three broad areas: 1) participants’ current and past positions and roles; 2) participants’ perceptions of the clientele they work with (youth and their families); and 3) participants’ understandings of how collaboration takes place within and between services and the community. We conducted inductive applied thematic analyses and then analyzed the interview transcripts of Inuit and non-Inuit participants separately to explore the similarities and differences in perceptions based on positionality.

**Results:**

We organized the findings around three themes: I) the most commonly described interventions, II) different types of challenges to and within participation; and III) what successful participation can look like according to service providers. Participants identified the challenges that families face in moving towards services as well as the challenges that services providers face in moving towards youth and families, including personal, organizational and historical factors.

**Conclusion:**

We adopt a critical lens to reflect on the key findings in order to tease out points of tension and paradoxes that might hinder the participation of youth and families, specifically in a social context of decolonization and self-governance of services.

## Background

The World Health Organization’s Declaration of Alma-Ata [1] and many researchers [2–4] call for governments and institutions to support the participation of citizens in both their personal clinical care as well as in the design and delivery of health and social services. Participation is understood here as a dynamic process centered on the active involvement of youth, their families, and service providers in care at the individual level as well as in services at the policy and program levels [2, 5–7]. A growing body of literature in the field of youth mental health suggests that young people from cultural minorities, including Indigenous youth, are less likely to seek help from health and social services and less likely to actively participate in decision-making regarding their treatment [8]. When youth do seek health services, it is often for more chronic and severe situations, and for shorter periods of time [8, 9]. This trend is not surprising considering the multiple historic and ongoing injustices that Indigenous people continue to experience within colonial health and social systems [10–12].

The disconnect between service provision and Indigenous youth is unfortunate considering the ways in which active patient participation in care can improve mental health promotion and prevention, decrease health disparities, and improve access to information [6, 13, 14]. Youth who are engaged in the design of their personal or collective care show increased self-esteem and are less likely to be involved in risky behaviors [15–18]. Youth and family participation within health and social services is particularly critical in intercultural contexts of social under-representation where the youth’s culture is not the dominant culture within the service setting [19, 20]. When youth participate in health and social services, care becomes more culturally appropriate and acceptable to youth and families, who then show improved access and adherence to existing services [20–24].

Service providers play an important role in establishing conditions that allow or encourage youth and families to move towards services and actively participate in care [25]. Through their approaches with youth and families, service providers can help develop the trust that is necessary to bridge the gap between communities and institutional services. These approaches will be highly influenced by service providers’ own working conditions and other organizational factors, [26, 27] as well as by their perspectives and expectations regarding the roles and responsibilities of the different actors involved in the wellbeing and care of youth. Understanding how service providers regard these roles and responsibilities, as well as the challenges and barriers they face in the context of their work is an important element of working towards conditions that support more active youth participation in care [28–32]. This article explores how Inuit and non-Inuit service providers who work with *Nunavimmiut* youth and their families perceive the roles and responsibilities as well as conditions for youth and family participation in services. *Nunavimmiut*, which means people of the land, refers to the Indigenous peoples whose ancestors lived in the Northern most regions of what is now called the province of Quebec, Canada. Before describing service providers’ perceptions and experiences, we begin by offering some context about health services in Nunavik and the needs that Inuit have previously expressed regarding services for youth and families. This context provides a frame of reference to then reflect on how the different and sometimes contradictory ways in which service providers understand youth, family and community participation in services, and how these understandings may influence the ability of the care system to achieve Inuit ways of knowing and doing within health and social care in Northern Quebec.

## Context

The region of Nunavik is home to approximately 13,000 *Nunavimmiut*. Ninety percent of Nunavik residents are Inuit. Nunavik is composed of 14 communities on two coasts, Hudson Bay and Ungava Bay, with community populations ranging from 200 to 2000 people [33]. Each community hosts a local Health and Social Service center, often referred to as ‘nursing station,’ that offers front-line medical and social services as well as youth protection services [11]. Although some individuals may approach social or child welfare services directly for support, many residents enter the health and social system via the nursing services. While the larger communities have permanent general physicians, the smaller communities receive visits from general practitioners for one week every month, and sometimes less often. Medical specialists including pediatricians, psychiatrists and dentists fly into specific communities periodically for assessments and follow-ups. Each coast has a hospital, such that there is one in Puvirnituq and one in Kuujjuaq, where people from the other communities are flown to access certain specialized services. For emergencies and specialized follow-ups, patients may be flown south to Montreal, which is approximately 1500 km away. Health and social services in Nunavik are funded by both the federal and provincial governments (approximately 30 and 70%, respectively) [34]. Services are under provincial legislation, which is different from First Nation communities in other parts of Canada, which are federally legislated [35, 36]. Whereas Inuit representation in service provision has historically been quite low, provincial laws in effect since 2012 have significantly diminished the possibilities for Inuit to work as front-line workers within institutional services [36, 37]. Inuit now hold positions as administrators, secretaries, interpreters, and community workers who generally accompany or translate for non-Inuit services providers. Leadership positions are open to Inuit, who can now take up positions as principals within schools or as department directors in the health and social service institutions. However, many front-line workers, and all providers who require professional accreditation (such as doctors, psychologists and dentists) remain non-Inuit staff [38].

Regarding Inuit experiences and expectations of services, a recent study conducted by the Nunavik Board of Health and Social Services suggests that Inuit generally feel satisfied with the services offered by their nursing stations and hospitals [39]. However, challenges remain, especially regarding mental health and social services for children, youth and families. A lack of preventative and front-line services leads to an over-reporting of families to youth protection services [40, 41]. Psychosocial difficulties experienced by children and youth can escalate quickly into crisis situations, and yet there are currently limited specialized services for such cases [42–44].

### Partnership research

In this context, the non-Inuit research team, led by a non-Inuit research professor, was invited to co-develop and seek funding for an action-oriented research program with local and regional partners in Nunavik to support community mobilization with youth and families. Through this program, we co-developed a community-led organization to support youth and family-oriented prevention activities. As part of this program, our local and regional partners wanted to better understand the existing institutional services and the experiences and needs of youth, families and service providers in order to better reflect on how to support the health and psychosocial needs of families. Our partners felt that this information would help to develop new strategies for improving community decision-making within community-led initiatives as well as within government-led health and social services. Specifically, this research could not only help to understand but also to transform services and improve youth participation in services within their community and within the region. Together we applied for funding for a series of different inter-related projects that would help map out the realities of the different people involved in the health and care of Inuit. The first set of studies focused on the experiences of Inuit families [44], Inuit community mobilisers [43] and Inuit service providers [45].

In one of our earlier studies [44], 14 Inuit parents described their perceptions of and experiences with health and social services. Parents spoke about the practices that they appreciated, as well as the barriers that they faced in accessing and using available services. Among these barriers, parents feared the consequences of using services because of their concerns about being reported to youth protection services or to the police. In this context, with limited prevention and front-line social services, parents identified that professionals and community members might signal families to youth protection as a way to ensure follow-up, regardless of the severity of a situation. In general, we found that families’ past experiences with the resources available in their communities influences their decisions regarding whether or not to continue seeking support when it is needed. Families’ perceptions of service providers’ abilities to be caring and non-judgmental influences families’ perceptions of the adequacy of care, which in turn influences their desire to seek support. Pro-active services, including home visits, were described positively.

In a later study, we worked with Inuit community members who are recognized for their effective work in health and social care in communities in order to identify Inuit practices and approaches in supporting youth and family wellness. Our key informants spoke about the values and objectives of health and social practices for *Nunavimmiut*. Here, care givers spoke about the importance of focusing on strengths, supporting individual and collective self-determination, upholding the interconnections between family and community, and how land and community are important locations for healing to take place (Gagnon-Dion M-H, Fraser SL, Louisa Cookie Brown: Inuit wellness: a better understanding of the principles that guide their actions and an overview of their practices, unpublished).

Then, working in partnership with a regional Inuit committee, our research team helped to design and then analyze community consultations about how communities wanted to see services transformed for youth and families. Following these consultations, the Inuk committee (informed by the results, described below) developed a framework for the creation of future services for youth and families. Our research team helped to organize this framework, which was constructed around six principles, including how children and families should be at the center of the design and delivery of all services, how Inuit should be the guides and decision-makers regarding all services grounded in Inuit knowledge and practices, and how the design and delivery of services should respect the realities and rhythms of Nunavik all the while supporting steps towards self-determination [45]. This iterative series of studies and consultations brought forth Inuit voices, experiences and requests regarding how service providers working within institutions could better meet their needs. With the motivation to move towards the self-governance of services, there is also a recognition that non-Inuit workers have much to contribute, as long as the work aligns with Inuit ways of knowing and doing. Yet community members recognized the gaps between institutional ways, and approaches desired by community.

This current study responds to the need to better understand this gap and to find ways to improve the current approaches and services, while Inuit work to develop their own systems of care. To do this, we draw on the work of Indigenous elder and scholar Willie Ermine [46], who articulated what he calls *ethical spaces of engagement*. Here, the intentions and experiences between Indigenous and non-Indigenous peoples have been blurred and complexified over centuries. Ethical practice requires an understanding of these experiences. By exploring the gaps and points of connection between institutional service providers and community members, it can become possible to reflect on ways for moving forward, towards services that are culturally relevant and guided by Inuit.

With a focus on ways to move forward, this study explores the realities within institutional health and social service systems and how these realities may influence the ability to put in place services that correspond to the Inuit principles, values and needs that were identified by community members in our previous research, above. We are particularly interested in the barriers and facilitators that are either explicitly expressed by service providers or implicit within their discourses. Interviews for this study were conducted with Inuit and non-Inuit service providers who work for different types of health and social service organizations, including schools, hospitals, nursing stations, youth protection services, and the police.

## Methods

The methods and interview guide for this study were co-developed with Inuit partners to capture their questions and interests. The interview guide was piloted with two participants and then reworked to ensure fluidity within the interviews. The project was submitted to the Nunavik Regional Board of Health and Social Services (NRBHSS), the Kativik School Board (KSB), now called Kativik Ilisarniliriniq (KI), and the first author’s university ethics review board for approval. To recruit participants, two non-Inuit research assistants worked with non-Inuit agents from the health board and from the school board to prepare a list of service providers who represented all “levels” (front-line workers, specialists and consultants, administrators, directors) of multiple health and social service organizations. The agents distributed a letter explaining the project to all service directors and school principals, and inviting their staff to participate. A snowball sampling method [47] was used to recruit 58 participants in three communities as well as in Montreal, including service providers residing in Montreal who fly in and out of communities for consultations.

A total of 54 interviews were conducted by the research assistants, who held Master’s level degrees in social sciences and had many years of experience conducting interviews in intercultural contexts. One research assistant had previously lived in Nunavik. Most of the interviews were conducted with individual participants, with the exception of four interviews that were conducted with two participants simultaneously, as requested by the participants. One non-Inuit individual refused to participate and stated that they generally did not feel comfortable with research. Participants included psychiatrists, general practitioners, nurses, social workers, school principals, teachers, student counsellors, representatives of local committees (such as an education committee and a health committee), and police officers. Of the 58 participants, 39 were non-Inuit and 19 were Inuit. Inuit participants worked primarily as either administrative planning agents or community workers (non-professional workers supporting social workers). Two Inuit participants were directors of services, one represented parents of children receiving intensive medical support, and three were community representatives working or volunteering for the community.

Interviews were conducted in the participants’ work area in English or in French and lasted approximately 90 min. The interviewers explained the context of the research as described above and how communities were reflecting on ways to develop community programs and services in order to better understand the existing services and actors involved in service provision, as well as the challenges and facilitators to health and social services in the region. The interviews then focused on three broad areas: 1) participants’ current and past positions/roles; 2) participants’ perceptions of the clientele they work with (youth and their families); and 3) participants’ understandings of how collaboration takes place within and between services and the community (who works with whom). All interviews were audio-recorded, transcribed, and subsequently analyzed using QDA Miner, a qualitative data software. Field notes were audio recorded and written by both research assistants.

Applied inductive thematic analyses were conducted by the non-Inuit research team. Thematic analysis allowed us to explore issues and experiences emerging in the data rather than according to pre-determined hypotheses [48]. The two first authors read through the entirety of the material and extracted initial themes and their related impressions. Our first impression of the data was the high presence of negativity. In order to determine whether this impression was a coder-bias or a phenomenon that was specific to certain groups of participants, we organized verbatim into positive, neutral and negative statements depending on the portrayed feelings. We then developed a large matrix to organize service providers’ descriptions of their relationships with other service providers, youth, families, extended families, and community members. We considered each of these relationships as a type of dyad. We conducted thematic analysis across each dyad to explore emerging categories, which included perceived roles, movement (actions) of collaboration, and where collaboration takes place. We then conducted thematic analysis within each category, which allowed us to explore the challenges and positive collaborations that were specific to each dyad. We also looked at the Inuit and non-Inuit transcripts separately in order to identify the specific experiences and perceptions of Inuit, which we focus our reflection on later in the discussion as a way to promote Inuit knowledge and self-determination. Making these distinctions between Inuit and non-Inuit also allowed us to explore the similarities and differences between the experiences and perceptions of community members and non-Inuit ‘outsiders.’ We selected participant quotations to represent each of the various themes and dynamics. To ensure participant anonymity, we made slight modifications to the quotations as well as to participant job titles.

Committed to partnership research, we note that this entire process was conducted over a 5-year period involving multiple action-oriented working sessions with the local committee, as well as with a newly formed regional Inuk committee [45]. The model presented in this article, and the reflections around the model were brought to both the community advisory board and the Regional Steering Committee for discussion and to inform decision making. While the decisions made by these two bodies are described elsewhere (46, 50), the results discussed in this article were written according to the principles developed in the model (see discussion for a complete list of the principles). We do not presume that this analysis captures the entirety of the experiences and perceptions shared by all participants, however offer the results as a model for prompting discussion and reflection.

## Results

First and foremost, we note that while both Inuit and non-Inuit health and social service providers often spoke about similar experiences regarding youth participation in services, they also had very distinct perceptions and expectations about these experiences based on their positionality. On the one hand, non-Inuit service providers tended to focus on the challenges they faced in engaging youth and families. Although non-Inuit expressed interest in engaging with extended family and community, their service provision practices often only integrated parents. On the other hand, Inuit service providers recognized certain challenges and also put forth many ideas about how to transform existing approaches and services. This focus on ‘how to move forward’ was striking. Moreover, Inuit spoke less about the actual services per se and more about the roles of different actors within service provision, in particular the role of community in supporting families and children.

We organized the findings about health and social service providers’ experiences and perceptions of youth participation in care around three themes: I) The most commonly described types of interventions; II) different types of challenges to and within youth and family participation; and III) what successful youth and family participation can look like, according to service providers. Inuit and non-Inuit voices are shared through-out the results.

### Commonly described interventions

In this section, we outline the most commonly described social interventions with youth and families. As mentioned above, service providers spoke predominantly of the difficulties they faced in engaging youth and families in services. We describe 1) the various actors discussed by participants and their perceived roles, and 2) their locations and movements between locations.

#### Actors and their perceived roles

Five core groups of actors emerged as essential partners with different roles and responsibilities for effectively providing services to youth and their families: a) service providers; b) youth; c) parents; d) extended families; and e) the community.

##### Service providers

Service providers saw themselves as having to share information with other professionals and having to communicate with parents in order to obtain consent to offer services to youth who are under the age of 14. Service providers felt that they often take the first steps in initiating contact and follow-up with youth and parents.

##### Youth

Despite youth’s central position in receiving care, service providers did not describe youth as having a particular role or responsibility. Youth are often described in relation to their behaviours, symptoms, family contexts and willingness to receive services.

##### Parents

Service providers described parental involvement as essential in the service delivery process, with parents sometimes described as potential coordinators of services. A non-Inuit doctor explained that parents can have a beneficial impact on the continuity and coherence of services as they can relay information from one service to another:

*“What goes best in pediatrics is when the parents are able to take on the role of coordinating the care, it's really when it goes well. Yeah, for coordinating and also for speaking for the child. Like, “You sent me for that specialist but that wasn't the one I needed. What I needed was this.” So, when there's that kind of empowerment and ability, then those really go best.”*

Service providers also described how parents can support professionals in finding solutions for youth. A non-Inuit teacher offered an example:

*“The parents came in for a meeting and we discussed the plan with the parents and [they] gave us their feedback about how it (the plan) would affect their children and some ideas were put forward”.*

##### Extended family

Service providers described the role of extended family members as support systems for parents when they are not physically or emotionally available. In fact, extended family is considered the first placement option when children must be removed from the immediate family environment. A non-Inuit Crown attorney emphasized how extended family can also be a source of information for service providers:

*“Family members will get involved most of the time, to help to find a solution for the child to be protected. Maybe they’ll take the child home and then this way the parents will maybe get a break for a while because sometimes it's difficult for them. And family members will also help me understand the situation a little bit better by giving me input.”*

##### Community

Finally, service providers often described “community” as a much needed collaborator for effective care, and more specifically for prevention. Service providers spoke about their interests in having community members guide their work and “mobilize” around health and social issues. An Inuk driver for a health clinic spoke about the supporting role that community must play for a young girl with behavioural issues:

*“She needs hope. How do we give hope? It's a community thing so we just need the people now to be better role models. We need to do our best and hope that she does her best, and that the community speaks to her and we need to hope that everyone will do their best (to help).”*

Another Inuk participant explained how communities can take on this leadership role:

*“There needs to be almost like a mission statement for the community. Like what, what do we want this community to be, what is it right now, where do we want to go with it? If we want to leave it status quo, by all means that is your right. But if we want to make it a little bit better, if we want to change it, if we’re not happy with the way it is, then let’s do something about it. And, that can’t just be from outside people coming from different universities and research and all, so it needs to come from the community members.”*

These words also serve as a reminder that research might be a tool for improving youth participation in services, but ultimately the process needs to be in the hands of Inuit.

Within commonly described interventions, these five groups of actors are reported as having distinct yet highly connected roles and responsibilities. In order for the service provider to work with the youth, they must interact with parents. For parents to be supportive towards their children and youth, service providers feel that parents need support from friends and extended family, who in turn require the support of community. However, services providers rarely described interacting with extended family or with community representatives or organisations.

#### Locations and movements

The physical locations of services and of those seeking services played an important role in service provider narratives about the role of youth participation in care. Children and youth often meet with service providers inside organisations such as youth protection offices, nursing stations, and schools. Parents were sometimes described as partners within service provision, although mostly as being difficult to reach and located outside of services.

Service providers used a variety of action verbs including *go to*, *come to*, *send*, and *call* that imply the need for movement when talking about their attempts to collaborate with families. Service providers generally described their movements and actions towards families and youth as their efforts to invite youth and parents to come and see them or to ask parents for consent to work with the youth. These efforts to contact families were conducted using email, letters, and phone calls, or sometimes by going directly to the family’s home. However, service providers also spoke about sometimes feeling uneasy going to people’s homes, especially if they know that the family is experiencing difficult psychosocial dynamics. At times, they would therefore ask a colleague from another service to accompany them to the home. They described these movements and actions *from* services *towards* community and would sometimes include individual consultations with particular members of the community, such as the mayor or an elder.

Regarding youth, very rarely did service providers describe active steps taken by youth to go towards services. Instead, youth were described as being “picked up” by service providers or “brought to” the services by another actor, often a professional. Referring to the example of suicide attempts among Inuit youth, a non-Inuit youth protection agent explained: *“But of their own will? Would youth come consult themselves? Teens? Because they aren’t doing well? No!* This same person explained that youth are often brought to services during a suicidal episode, and according to her perceptions at least three quarters of these youth will not continue with the proposed follow-up. Similarly, an Inuk rehabilitation officer explained:

“*The parents don't usually call me. When I meet them, it’s because their son or daughter has been arrested. And then that's when they're going to say, “can you help my child, can you try to convince him to listen to me, to go back to school … ?”*”

In general, youth seem to be understood as passive agents within health and social service interactions. Many non-Inuit service providers placed a stronger emphasis on the role of parents. While the importance and potential of other actors such as extended family and community are recognized by service providers, there seems to be a disconnect between the locations of services and the locations of actors, and how agency and who is moving towards whom are perceived.

### Challenges to and within encounters

Service providers’ narratives about youth involvement in care emphasized two inter-related challenges to making connections with youth and families: 1) Challenges that inhibit the use of services by youth and families (as understood by service providers); and 2) Challenges that impact service providers’ ability and desire to go towards youth, families and communities.

#### Inhibitors to going towards services, according to service providers

In this section, we identify four broad factors that influence families’ use and perception of services: a) colonialism; b) service provider’s attitudes; c) fear, stigma and discomfort; and d) limited service mandates.

##### Colonialism

For Inuit participants, historical considerations were more prominent in their explanations of difficult encounters. Many spoke about the impact of forced sedentarisation on families as well as intergenerational trauma, alluding to the need for collective healing in order to deal with the traumatic issues that have been passed from generation to generation, beginning with the years when Inuit were forced to be sedentary. Only a few non-Inuit participants identified how colonial histories might influence how families interact with services. A non-Inuit nurse explained the irony of the colonial situation that Inuit must contend with:

*“[Inuit] lived in igloos and they had their traditional way of life, and then we (non-Inuit) came in and said we're going to give you those villages and we're going to kill your dogs. We're going to force a different kind of food on you and we're going to basically manage you the way we want to. Then we're going to put you in schools, where a lot of you are going to get abused and whatnot. Then suddenly we're in 2014 and we're asking: How come you guys are not taking care of you own life?”*

This nurse described a feeling of frustration and disempowerment in the larger social context where Inuit families are being asked to trust services and mobilize healthcare plans produced within a colonial system.

##### Service provider attitudes

Both Inuit and non-Inuit participants acknowledged how service providers’ attitudes influence whether or not youth and families will consider services as acceptable. Some service providers described negative, and at times hostile, attitudes amongst their colleagues who act in ways that enact or reinforce colonial relations. These discriminatory attitudes can directly influence families’ abilities to trust the services. An Inuk parent described the discrimination that their family experienced, when they were told by a doctor that if they missed an appointment for their child who was dealing with an important medical condition that the nursing station would contact youth protection services. After denouncing the doctor, the parents never heard back from her. These experiences of discrimination fuel existing mistrust.

A non-Inuit family doctor at the hospital described some of the judgmental attitudes that she has observed among her colleagues, which she believes may impact families’ comfort in using services:

*“I find there are lots of people who judge quickly … Like, my child is half Inuk and she says that when she goes to the hospital, if she is with me, she sees a difference in how she is treated. When she isn’t with me, she says that they don’t always treat her nicely. I feel there can be discrimination.”*

Some participants also felt that certain service providers interpret and label behaviours as ‘cultural ways,’ rather than truly attempting to understand the uniqueness of a person, and the complexities and contexts within which individual actions take place.

*“Some workers make conclusions like, “ahhh it's because of their culture, she can't tell me this or she can't talk.” I don't know what it is but with the whole White and Inuit … I'm so over it. That's enough (blaming culture). It's the 21st century - we're gonna work together or we're not? It's time to work together, everybody. Stop blaming culture.*

Indeed, these statements are not uncommon in Nunavik; non-Inuit workers feel frustrated about not being able to connect with Inuit families. Interpreting these challenges as cultural differences removes any possibilities of transforming one’s own practice, or of understanding tensions in a different light.

##### Families’ fear of services, stigma associated with service use, and feelings of discomfort

Some non-Inuit service providers thought that families might see services as a form of punishment rather than as a source of support. They also felt that for some parents, seeking help through services can be stigmatizing within their community. Other parents may fear service providers taking away their children or the police getting involved in their family life. A non-Inuit child psychiatrist gave an example of a family dealing with this fear:

*“The mother was very traumatized by the DYP (Department of Youth Protection), so she will stay away from the ‘medical’ (services) as much as possible; basically, all that is ‘White.’ It's a shame because the children … they need help, they have a lot of learning difficulties and then they (families) go to look for help. Sometimes a mother accepts, then she withdraws because she is so afraid … she remains scared that her children will all be removed again.”*

A non-Inuit social worker described how parents might feel guilty when a service provider or teacher approaches them with a situation concerning their child, which can lead to distancing themselves from the service providers:

***“****Well, if there is any [problem], there is tension with the family. If your child is not doing well at school, the parent feels guilty about everything, and then they close down.”*

A non-Inuit crisis center coordinator felt that families might fear being judged by other community members by accessing particular services, for example, related to mental health.

Inuit workers remind us that the position of being a youth in society can hinder their desire and ability to get help. This position is then compounded by the fact that workers are mostly non-Inuit who youth do not know and therefore feel even less comfortable seeking help or opening up. One participant described how this can lead to crisis situations: “*They close up because the workers are from another culture. They keep it all in, they need to let it out... It just explodes.*”

##### Service mandates

Several service providers also described how different understandings of the role and mandate of various services could impact how patients access and use these services. A non-Inuit mental health nurse shared how he responded to a situation when a youth misunderstood the role of his youth protection worker:

*“Often they don’t understand. Like for example, I was following a youth under DYP. The youth verbalised that he hated his DYP worker, but he didn’t understand her role at all, what she was doing for him. Sometimes I spent time with him, telling him: Listen she wants what is best for you, she is there to ensure this, that and that. She wants to help you go back to school.”*

An Inuk worker explained that there may be services and activities within the community for youth and families but that if people do not know about these activities or do not understand why and where they are taking place, then people will not attend. Some participants encouraged using direct invitations to activities or community radio as a means of communication.

From the perspectives of service providers, the factors that inhibit families’ use of services—such as colonial histories, service provider attitudes, families fear of services, and misunderstandings about service provision mandates—create barriers from families ‘moving towards’ or accessing services. At the same time, a range of factors also inhibit how service providers engage with youth and families.

#### Factors that influence service providers’ abilities and desires to go towards youth and families

Service providers identified six factors that constrain them from reaching out to and engaging with youth and families: a) Parental consent; b) a lack of resources within the community; c) language; d) culture; e) mismatched timing; and f) challenges of being from the community.

##### Parental consent

Participants spoke of legal challenges to genuinely engaging youth and families in service provision. In order to provide services to youth under the age of 14, parental consent is legally required. Consent is also required to share information with other service providers. Service providers described a strained dynamic where they either feel dependent on receiving consent from parents in order to provide services to youth, or instead chose to use the institutional power of youth protection services to oblige service provision. A non-Inuit social worker from the nursing station, who also worked at a local school, described the challenges to obtaining parental consent:

*“I always try to get consent from parents, especially when the youth is under 14 and well, sometimes they refuse. You cut the grass under my feet, I can’t do anything. Sometimes I work in collaboration with DYP and then they might be able to get a consent from parents after trying very hard. I have to send a paper, they have to sign it, and then I never see the parents again. They sign, I have the paper … we invite parents to come meet, again with pressure from youth protection, and often the parents won’t show up.”*

Consent is essential in order to ensure parents are decision-makers in a process of care for their children, however consent requirements also seem to construct and formalize particular types of relationships between service providers and families.

##### A lack of resources within the community

Service providers perceived a lack of resources as a challenge to setting up alternative services that would better respond to community needs, for example, related to emergency housing, in-community alcohol and drug rehabilitation services, psychotherapy, and financial assistance. A non-Inuit youth protection worker, who specialises in clinical care, explained how the lack of resources for children who are signalled under youth protection directly impacts the chain and quality of services that health workers can provide for youth:

*“There is a lot of placement and there are very specific protocols and frameworks about when to put a child in and what to try, how to do it, and how to prioritize and what to do with it … The law can be rigid. But here, unfortunately, we do not have foster families. So, we end up placing [youth] in places that are not necessarily better or placing them with Whites who will eventually go one day. And I do not judge, but that's it anyway. So, we take children, we take them away [from their families] and they lose all contact because the Whites who speak Inuktitut are not many.”*

Service providers, like this youth protection worker, described feeling frustrated and discouraged that they do not have more adequate resources to develop and implement comprehensive solutions and plans that better meet the needs of the youth and family they work with.

Similarly, Inuit workers described feeling irritated with the types of services offered in their community, and how the low number of human resources compared to the needs greatly influences the nature of services. An Inuk service provider explained:

*“I totally know and understand what [non-Inuit workers’] situation is about, how overwhelmed they are … but you know a lot of the time, they don’t set themselves up for success either. I just know for a fact that all these people are so overwhelmed because there’s a giant workload as soon as they come in to work and it’s hard for them to keep up. It’s like everyone is just thrown under the bus. So, there’s no time for them to think about prevention, they don’t have the time to think about counselling, they don’t have time to just do recreational activities.”*

##### Language

Non-Inuit participants identified language as a major impediment to developing positive interactions with families. Communication challenges seem to create frustrations for both service providers and family members who feel that their exchanges are limited when they would like to engage further. A non-Inuit psychoeducator explained:

*“One of the problems I have, it's Inuktitut. I do not speak Inuktitut … When you have young people, when you get into the emotions, it's all in Inuktitut. They spit it out (in Inuktitut), and you would have to understand what is said. There are young people who know I do not speak Inuktitut, but sometimes I get a sentence in Inuktitut and they are discouraged that they do not know how to say it in French or in English*.”

While non-Inuit service providers might sometimes learn a few basic words of Inuktitut, rarely do they have a working knowledge of the language. Moreover, for many service providers, English is a second language. In these cases, both the family and the service provider are exchanging in a second language. This can be challenging and tedious in any situation, but particularly when speaking about emotions and relationships. As will be described below, language is yet another reason for non-Inuit workers to work in collaboration with Inuit workers or other community members.

##### Culture

Some service providers talked about the ambivalence and complexities related to non-Inuit learning about and from Inuit culture. On one hand, some participants remarked that these efforts may be perceived positively, as a form of respect. On the other hand, participants suggested that these efforts can also be perceived negatively, as “wanting to be Inuit.” Participants also described how community members can limit non-Inuit access to cultural activities or how community members might limit their general interactions with non-Inuit individuals who are perceived as not being authentic in their attempts to learn or as attempting to appropriate traditional activities. A non-Inuit nurse explained:

*“There are some (non-Inuit) who will be able to speak Inuktitut … They always come up with Inuktitut sentences in the meetings. Then Inuit will tell me: damn they annoy us ... But it was only after a few years that I started hearing that. In the beginning you think ‘I have to become like that, I have to.’ But now, collaboration for me ... It's about being yourself.”*

Cultural challenges also emerge when people have different and often incompatible expectations of how youth spend their time. A non-Inuit social worker offered examples such as school teachers expecting youth to attend classes every day and all day, and social workers hoping that youth will attend prevention sessions on a regular basis, whereas families might feel that activities such as hunting, camping or staying at home are most helpful for the youth. Inuit participants discussed the importance of cultural sensitivity training for non-Inuit workers and an interest in integrating Inuit and non-Inuit workers in the same training so as to ensure shared learning and to improve the ease of working together.

##### Mismatched timing

From the perspectives of service providers, families use services at times and in ways that are inconsistent with the ways that services are typically delivered. Indeed, service providers reported that families often ask for help when they are in a precarious situation. However, because of the lack of resources in communities, families often only receive help when the situation becomes critical. A planning officer at Youth and Family Services described the situation of a family who had been asking youth protection services for support because they were concerned that their teenager was engaging in drug use and sexualized behaviors, yet they did not receive any services. In a moment of crisis, one family member hit the teenager. Youth protection services then got involved and placed the child in foster care. In another example shared by an intervention worker, a parent called the police to ask for help in dealing with their teenager who was heavily intoxicated. Yet the police did not see themselves as having a mandate or a role in this situation. In these two examples, families reached out for help but could not access the services at the moments when they were needed the most.

Furthermore, many service providers described their impression that in times of crisis, families expected services to take charge of a situation and of their children, relegating their parental responsibilities entirely. A non-Inuit psychiatrist described how youth can end up hospitalised alone in Montreal:

*“Sometimes, youth that are hospitalised - their parents don’t come to see them. We have to run after the parents. The social services try to reach the parents. The youth is a minor and doesn’t have family around. We have extended family who might be there a bit and that is really helpful. Or else, they end up alone.”*

In a contrasting example, a non-Inuit social worker explained how families might show up in times of crisis:

*“People call when they are having a big issue, big distress, crises … when they are really upset. They aren’t able to keep their child, not able to keep their elderly parent, or not able to deal with alcohol problems of a family member. It’s pretty much what we deal with. Yes, we offer support, but it stops there. Because if people don’t take things into their own hands, well the problem just starts all over again. Me, I try to show the cycle of dependence. I try to show ways out, ways of affirming oneself, how to face our own problems.”*

Family members may seek services on different occasions or may stay at home feeling that the resources are not helpful. If the situation spirals into a crisis, families may either feel the need to go back for support or end up forcefully receiving court-ordered services. This spiral has multiple repercussions. In the moment when services are offered or available, the family may have already fallen into feelings of hopelessness and disengagement towards the situation.

Service providers also spoke of feeling frustrated when situations ended in crisis when they thought that the crisis could have been prevented. This frustration was at times accentuated by service provider attitudes and assumptions about Inuit. For example, a non-Inuit service worker articulated the stereotype that Inuit are not ‘prevention oriented’:

*“In general, health services are very well received by the population. Typically, the Inuit population is a population that lives from day to day. So, when we talk about curative health care, yes, they are engaged, they come to seek this care. Less when we talk about prevention. It is not necessarily a population that will be compliant with prevention programs or come for their medical appointments. If it is beautiful that day, they will go fishing and then hunt. They will not come to their appointments..”*

These stereotypes and frustrations may be felt and heard by other service providers as well as by the families who may feel judged or misunderstood. An Inuit elder explained the challenges of navigating obscure bureaucracies and of having a genuine community voice within services:

*“Even if we meet and talk and say what we need as a people, there are too many other things that influence decision-making, things we cannot see. So, in the end we don’t feel heard, we don’t feel understood. What is the point?”*

Mismatched timings between the moments when services are needed (before the breaking point) and the moments when services are offered (at the breaking point), as well as the tendencies towards making generalizations and assumptions, seems to impact both service providers’ and families’ perceptions of one another, limiting their abilities to collaborate effectively towards a common goal.

##### Working and being from the community

Both Inuit and non-Inuit workers talked about the challenges that Inuit workers face when working in their own community. Working with youth and parents who are also relatives or neighbors can be socially and professionally complicated. Inuit workers felt that they should be offered counselling and guidance in their work to help deal with these realities. They felt that the lack of social support and counselling inhibits their ability to consistently provide the care they would want to offer to their community. An Inuk worker explained:

*“You know what, I worked out of passion, out of love and I did this for my community. I felt like I was making a positive impact, and then my friend (who was also working for community services) said, ‘it wasn’t worth being shut-out (by community member) for $15 an hour.’ It wasn’t worth family disowning them, or not feeling safe to go in public. I swear it blows my mind … After I was at the TRC [Truth and Reconciliation Commission] last year, I felt and saw how really deep everything is, and how my generation and a little bit older, are affected. I have suggested in the past to have counselling, a counsellor for the counsellors. That or intercommunity counselling. Like, let’s say I’m a social worker in this community, I’m having a really hard time [and] I don’t feel like I’m being welcomed by my community. I need to be able to speak to someone about it.”*

Both Inuit and non-Inuit service providers described a range of factors that affect their ability and desire to moving youth and families. Service providers might speak to each actor individually but rarely together, and services providers and family members might occupy distinct spaces within the community that make it difficult to meet. From seemingly procedural factors such as the bureaucracy around consent, miscommunications around language, and mismatched timing around when services are requested or needed and ultimately offered, to deeper structural issues related to a lack of resources within communities and culture, each of these areas represent barriers or hurdles to youth and family participation, as perceived by service providers.

### Building on strengths

Despite the multiple challenges above, many Inuit and a few non-Inuit service providers described successful encounters with youth and families as well as the specific ingredients or approaches that they felt would contribute to more positive and meaningful participation of youth families in care. We have organized these success factors around five categories: a) developing trusting relationships; b) informal encounters; c) reaching out to extended family and community; d) responding to the right needs; and e) attitude and care from service providers. We note that Inuit participants specifically spoke of trusting relationships and the role of community members in supporting the wellbeing of youth. Inuit also talked about ways of training non-Inuit service providers to improve cultural awareness and attitudes. Inuit workers, who are also parents and community members receiving services, looked beyond their disappointments with the current situation and identified possibilities for transforming approaches and structures of care. Here, we integrate Inuit and non-Inuit perspectives to reflect on ways of learning from positive experiences and building on existing strengths, while always basing the categories around how Inuit within this study and in past studies have suggested to improve care.

#### Developing trusting relationships

Non-Inuit participants spoke of service providers who have lived in community for many years and who have established trusting relationships with families. They felt that when this was the case, families would mobilize in their care and proactively seek help. A non-Inuit child psychiatrist described how her ongoing relationship with and commitment to the community helps build trust and collaboration:

*“I've been there for a few years. [Families are] starting to recognize me, they greet me. Then they'll come to the airport and then they'll tell me "you know my daughter, such, such thing." It is in the long term that the relationship is established and then the collaboration is done.”*

After having received training from an Indigenous organisation, a non-Inuit nurse reflected on what she learned through the training:

*“Often when Inuit go towards services, it’s because they are in crisis and they just don’t have any other choice. But would they really just go when they are in crisis if they felt that they had strong trusting relationships and if services were adapted? I don’t think so because I know people who have good relationships with workers, and they don’t just go when they are in crisis.”*

When a relationship has been difficult to establish with a patient or family, some service providers described building on another service provider’s trusting relationship by asking the colleague to speak with the family for them or to accompany them in their meeting with the family. This approach was particularly relevant with Inuit colleagues. For example, an Inuk youth protection worker described how she has collaborated with her non-Inuit colleagues:

*“Literally for every intervention. I heard so many [of my colleagues], like “it’s impossible to get to this mother, it’s impossible to go to this house” ‘cause they are alone, you know? Like, we discussed before, you should have an Inuk with you every time you go to someone’s house. So, I follow to people's houses just to go translate and it usually works.”*

Another Inuit psychosocial worker described playing a similar role:

*“When I was in charge of this service, we had caseloads and we had waiting lists and everything. But we could work much faster and quicker with Inuit families because we're Inuit. I had an assistant who was also Inuit, and elderly experienced people working with us as community workers and going to visit families and knocking on doors and working with the police. So, it was much more community-oriented.”*

#### Community involvement and informal encounters

Inuit workers talked about wishing that non-Inuit workers would get more involved in the community to get to know the families and create that trust that is essential for clinical care. One Inuit service provider said:

*“In my mind, as a front-line worker and a community member, the way a community member will see a front-line worker is only through when there is a crisis. So, a front-line worker in the community is a crisis intervener and then it’s not always positive. So, I would love them to be more involved in the community. It can be by volunteering, you know, coming in to our recreation facility and volunteering … you know, play soccer or be a part of a sports team.”*

Non-Inuit participants who described positive collaborations with families spoke of using informal.

approaches such as “having an open door to just come and meet.” A non-Inuit social worker described what this open door could potentially look like:

*“There’s got to be an open door. You know, I was even discussing with a couple of the local staff in the school - how cool would it be if once a month, we just had like an open-door night for a few hours? Like, not parent-teacher night, not talking about report cards … we are not discussing the academics. Just come and meet the teacher. Come have a coffee, relax, you know? It doesn’t have to be fancy, it doesn’t have to be organised. The idea that the teacher is not this scary entity that sits in a classroom, right? That we are human beings and we’re just here to help your kids.”*

A non-Inuit teacher explained that he tends to spend a lot of time within the community, at events and just walking around chatting with people:

*“I guess it's also about being in the community a fair amount, so I am very close with a lot of parents. Like, I know them on a social basis. And I'll talk to them about their kids at those points, too. I think it helps sometimes.”*

#### Reaching out to extended family and community

Working with extended family members such as grandparents, aunts and uncles, and cousins as well as key members of the community can also help service providers connect with the family and youth. A non-Inuit general practitioner explained:

*“[The] times I've seen beautiful interventions were often [with] the grandparents who know the children well, and the grandparents really have a respect here from young offenders. I think it's often them who have a lever to try to reason youth and talk to them about more emotional things.”*

A non-Inuit psychiatrist explained the particular involvement of extended family in Nunavik:

*“I find that compared to the south, the extended families really get very involved with patients … Aunts, cousins, there is really this sense of family that goes beyond the nuclear family. And generally, it goes pretty well with them when they see that we are interested in them, that their opinions count, that we are soliciting them for that, when we thank them for their support for example. They are often really pleased. They have a collaborative mindset”.*

A few participants spoke about these types of consultations yet admitted only seeking this wider community engagement once or twice during their time in the north. More frequently, service providers encouraged patients to consult extended family as well as members of the clergy. A non-Inuit public health agent explained:

*“When I started working in my two regular villages, I went to see the Mayor of each community to ask what he saw, as a leader, what were the main problems. And what he thought could be done to adapt service more to their culture. I did it at the very beginning of my practice. I was well received each time. The Mayors were very grateful. But after that, I never did it again”.*

Other non-Inuit individuals, including a program manager, a planning officer and a school director, described trying to “go towards community” as a way to learn from community and integrate the environments they live and work in. They talked about trying to learn Inuktitut or traditional activities, spending time on the land, participating in community activities, and integrating themselves into the community. These efforts align with how an Inuk participant explained that workers can be pro-active in their support to families by going directly to their homes and actively looking for solutions with the family:

*“I have been trying to volunteer … like (running) a workshop, like to train parents by doing home visits, just teach them the basic needs and what they need at home. It's a lot of fun because it's a lot of interactions and some parents, they don't know what to do so it would be fun to have home visits. Talk about what kind of support they need, not judgemental.”*

#### Attitude and care

Finally, service providers spoke of how attitudes of respect and care for families can allow for positive interventions built on trust. A non-Inuit doctor explained the importance of recognizing the role that families play in patient care:

*“To feel that [service providers are] interested in what [families] have to say, that their opinion counts, that they have been asked for that, that they are thanked for the support they give for the patient, for example. They are often very happy with that.”*

In the quotation below, a non-Inuit psychosocial worker recounted the story of a foster family and youth protection agent who respected the importance of the mother in the child’s life, despite her challenges:

*“The baby is placed in foster care in the South, but this foster family is so available and open. They text [the mother] and they talk to each other every day [on] Facebook, so that the mother can keep a link with [her] baby … who is 2 years old. But this foster family there, so available. Then the social worker who works with her, she is very aware about the reality of First Nations, the importance of maintaining links, culture.*

While messaging through social media is certainly a limited form of contact, in the very difficult situation of foster care, where many parents often lose contact with their children completely, this commitment to the Inuit mother was an important example of care for the service provider.

#### Rethinking the role of community

Inuit workers who participated in the study spoke mostly of the role of community in supporting youth and families, and how community members with life experience building trusting relationships as a way of teaching and engaging youth. They also talked about the importance of spaces in the community where youth and families can come together with workers to do cultural activities and spend quality time together. These spaces and activities were seen as the foundation for wellness and a way to create connections with service providers who can offer clinical help. An Inuk complaints commissioner gave the example of a community kitchen:

*“There is a community kitchen that happens every Monday, Wednesday, Friday. Three times a week, going on here at school for the whole family. Sometimes I bring my children and then you can bring your children; they have animators for the children. I think little things like that can help relationships between parents and young people.”*

## Discussion: shifting how service providers see participation

Inuit of Nunavik are currently in the process of transforming and developing services for youth and families [45]. The vision is one of self-determination with Inuit as guides and decision-makers in the design and delivery of services, and Inuit creating the foundation of these services. Yet there is also the recognition that non-Inuit and the institutions in place play an important role in the care of youth and families [45]. Therefore, in this period of transition towards self-determination, Inuit and non-Inuit wonder how service providers within the existing services can transform their practices to enhance the participation of youth and families in health and social services. As previous studies have shown, Inuit have shared their desire for more community-led practices, pro-active in-home approaches, and ‘beyond-mandate’ service provision for families [44, 45, 49]. Inuit have asked for more prevention-oriented approaches that are grounded in Inuit knowledge and ways and that bring families and communities together rather than treating patients or clients as individuals [44] (Gagnon-Dion M-H, Fraser S.L. & Louisa Cookie Brown: Inuit wellness: a better understanding of the principles that guide their actions and an overview of their practices, unpublished). In this discussion, we reflect on these principles and values and discuss how the experiences of Inuit and non-Inuit service providers can shed light on both the tensions that might hinder the ability to achieve these goals, as well as promising opportunities for change.

While both Inuit and non-Inuit described a variety of challenges and promising practices for supporting the participation of youth and families in their health and social care, exploring Inuit and non-Inuit narratives separately has highlighted two important differences. First, non-Inuit generally tended to replicate more negative discourses about the limitations to participation in care such as not having enough resources and time. Although not always the case, many non-Inuit workers described difficult experiences and feelings of frustration, disempowerment, and feeling stuck. Unfortunately, these narratives sometimes fuel stereotyping, discrimination and negative attitudes, for example, around Inuit as not being prevention oriented, or not being engaged in the ways that service providers want. Second, Inuit tended to offer hope and ways of moving forward towards greater self-determination in relation to youth care. Even if Inuit participants are both community members and workers within the systems, they described promising practices that are very much grounded within community, as opposed to within the institutional services. Inuit see communities as spaces for connecting, learning, and healing. Communities are also seen as potential decision-makers. There is a clear invitation for non-Inuit service providers to be more present in community life and to learn from Inuit to create trusting relationships.

Next, we discuss the content of participants’ narratives around three essential components of psychosocial practices as elaborated by Inuit in previous research: understanding historical considerations; being grounded in community; and focusing on kinship [44, 45, 49] (Gagnon-Dion M-H, Fraser S.L. & Louisa Cookie Brown: Inuit wellness: a better understanding of the principles that guide their actions and an overview of their practices, unpublished).

First, despite the importance of colonization and colonialism in the development of negative attitudes [12, 50], very few service providers spoke about history and its potential impact on youth and families. Colonization and the associated traumas, including loss and grief, separation of families and children, the taking away of land, the loss of culture and identity, and the resulting social inequities, continue to affect the way youth and families perceive services and interact with them [20, 51]. The persistent remnants of colonial history can infiltrate the interactions between health and social service providers and youth and their families, leading to difficulties in building trusting relationships [23]. In our view, understanding history offers a critical entry point and possibility for building relationships and for service providers to recognize the importance of culture, language, identity and place.

Second, non-Inuit service providers difficulties in reaching out to youth are related to the location and mandates within services. As Inuit service providers in this study, and community members in previous studies (Gagnon-Dion M-H, Fraser S.L. & Louisa Cookie Brown: Inuit wellness: a better understanding of the principles that guide their actions and an overview of their practices, unpublished), have explained, there are immense possibilities for supporting youth and families within the communities themselves. Various Indigenous authors remind us that community is the space where people can gain deeper insight into the hearts and realities of Indigenous peoples and therefore the service providers who wish to bridge this gap must integrate community to connect with people [8, 51, 52]. For example, Vicary and Westerman [51] showed that Indigenous participants preferred a non-Indigenous therapist who was interested in developing a *holistic* relationship with them that was not limited to professional settings. Inviting participation is not an event such as a phone call or an email; it is a bond and an open attitude. When connections to place, how people relate to particular social and cultural spaces and places, are respected and strengthened, community participation in services can be improved [53–55]. Our findings align with research exploring ways of engaging Indigenous youth in health and social services, which highlights the importance of reaching out to youth and meeting youth in their environments, perhaps with their families, and on their own time through informal interactions and trust building [8, 20, 51, 56]. This is important to reflect upon. If service providers expect families to move towards services and participate within institutional boundaries, then the absence of family members within the walls of the nursing station can be frustrating for the service providers. If community is seen as a space for trust building and healing with youth and families, then service providers must be encouraged and supported in transforming their protocols and approaches. A few non-Inuit participants in this study suggested that positive encounters are often described as informal, which means that service provider mandates and attitudes must include being flexible, spontaneous and open to meet and discuss outside of the institutional spaces and outside of office hours. This current study identifies barriers related to space, time, mandate and a lack of resources, and how the positions of various actors within specific geographical locations can either enable and limit youth participation in health and social care.

However, for all of this to take place and as Inuit participants tell us, health and social services require major financial and structural transformation. Similarly, Campbell and Erbstein [52] highlighted the need for greater time and resources for intervention, for cultural changes within organizations, and for the development of particular values that underpin leadership, such as community rootedness, relationships, knowledge, and legitimacy among the staff. Adapting services to Indigenous needs and culture, particularly of youth, often requires extending beyond existing service mandates [52].

Third, Inuit have expressed the need to work in ways that respect Inuit kinship and that strengthen interconnections [45] (Gagnon-Dion M-H, Fraser S.L. & Louisa Cookie Brown: Inuit wellness: a better understanding of the principles that guide their actions and an overview of their practices, unpublished). Within the current study, service providers also identified a multitude of interdependent actors that play important roles in the care of children and youth. These findings expand the literature on collaborative care, patient-centered care, health coalitions, and youth engagement, which each tend to focus on more narrow relations, often between two groups of actors such as service providers connecting with patients, different types of health professionals connecting amongst themselves [57, 58], and community organizations connecting with institutional (formal) organizations [59]. The service providers interviewed in this study suggested that youth are connected to a variety of actors (including parents, extended family, and community) who play distinct roles, and who are co-dependent in the care of youth. However, our findings suggest that these groups and individuals may not have the opportunities to all interact with one-another due to structural realities or organizational cultures. For example, by focusing on parental consent to work with children, as well as on notions of confidentiality, health service providers may have difficulty building partnerships with parents and extended families. Moreover, with limited human resources and many families needing health and social services, service providers end up focused on crisis interventions rather than prevention and support programming. Participants who did talk about being able to connect with families described the importance of working with Inuit workers, cultural consultants, or at least, non-Indigenous professionals that have an established trusting relationships with the community over the years. Interestingly, in a study conducted in Australia, Vicary [60] found that 92% of Aboriginal participants would not see a non-Indigenous service provider unless a cultural consultant recommended the service provider to them. Westerman [8] adds to this literature by suggesting that engaging a cultural consultant in service provision has to be done in manner that is coherent with culture and beliefs. It is important to note that the current study was conducted primarily by non-Inuit researchers. Despite having developed strong relationships with community members and having held various brainstorming and planning sessions with Inuit, the way of organising and sharing information in this article remains highly influenced by Western ways of seeing and doing. Moreover, the interviews were conducted with service providers who were interested in speaking about their experiences and perceptions. This most likely homogenizes the voices of service providers within the current study. The interviews were conducted in English by non-Inuit research assistants who were not connected with community at that time. Although they have continued to be heavily involved in partnership research and Inuit-led initiatives since the interviews took place, the fact that they did not have long standing relationships with Inuit at the time may have limited the number of interviews conducted with Inuit. For all of the reasons, and those stated within the present article, Inuit workers and families should always be seen as the guides to their health and social care.

## Conclusion

This study highlighted health and social service practices that are viewed by service providers as being helpful in creating connections with youth and families. Service providers, and more specifically Inuit service providers, emphasized the importance of sensitivity to colonization, and understanding the ways in which colonialism and its impacts are ongoing. Non-Inuit workers encourage service providers to explore ‘out of the box’ approaches that include asking Inuit for guidance, collaborating with Inuit workers, building relationships within the community, and advocating for structural transformations as requested by Inuit. It would seem however that many structural issues can impede these transformations in approaches to service provision [8, 52, 61]. Non-Inuit and Inuit service providers might feel stuck, with limited flexibility within service institutions. These various personal and organisational limitations might lead to feelings of frustration towards the system or towards the families they work with. For Inuit participants, communities seem to be spaces of opportunities. In order to reduce the gap between services and families, significant changes must be made so that Inuit can be decision-makers about approaches to services and help to guide non-Inuit towards approaches that are supportive. Therefore, rather than asking how we can increase youth and family participation in health and social services, we propose a shift in perspective with the following question: How can we create spaces and processes for service providers to better see and support the existing participation of family members in the design and delivery of care? And how can we better listen to Inuit families and service providers who clearly have ideas of how to transform services and approaches?

## Data Availability

The datasets generated and analysed during the current study are not publicly available due to the confidential nature of the information that is shared in interviews conducted in a small region where people could be recognizable. However, the data could be made available from the corresponding author on reasonable request and with the approval of the Nunavik Regional Board of Health and Social Services.
